# Prognostic value of different maternal obesity phenotypes in predicting offspring obesity in a family-based cohort study

**DOI:** 10.1186/s12889-021-10932-4

**Published:** 2021-05-08

**Authors:** Sara Jalali-Farahani, Parisa Amiri, Bita Lashkari, Leila Cheraghi, Farhad Hosseinpanah, Fereidoun Azizi

**Affiliations:** 1 Research Center for Social Determinants of Health, Research Institute for Endocrine Sciences, Shahid Beheshti University of Medical Sciences, P.O.Box: 19395-4763, Tehran, Iran; 2 Students’ Research Committee, Shahid Beheshti University of Medical Sciences, Tehran, Iran; 3 Department of Epidemiology and Biostatistics, Research Institute for Endocrine Sciences, Shahid Beheshti University of Medical Sciences, Tehran, Iran; 4 Obesity Research Center, Research Institute for Endocrine Sciences, Shahid Beheshti University of Medical Sciences, Tehran, Iran; 5 Endocrine Research Center, Research Institute for Endocrine Sciences, Shahid Beheshti University of Medical Sciences, Tehran, Iran

**Keywords:** Obesity, Children, Obesity phenotype, Mother, Incidence

## Abstract

**Background:**

Parental weight is studied as an important determinant of childhood obesity; however, obesity-related metabolic abnormalities have been less considered as determinants of childhood obesity. This study aimed to investigate the association between maternal obesity phenotypes and incidence of obesity in their offspring.

**Methods:**

This longitudinal study was conducted within the framework of the Tehran Lipid and Glucose Study. A total of 2151 non-obese children who had complete parental information were followed for incidence of obesity over a mean of 148.7 ± 34.7 months. Obesity in children was defined using the World Health Organization criteria. Maternal body mass index (BMI) was classified into three categories: normal weight, overweight and obese. Dysmetabolic status was considered as having metabolic syndrome or diabetes. Metabolic syndrome and diabetes were defined according to the Joint Interim Statement and American diabetes association criteria, respectively. Considering maternal BMI categories and metabolic status, six obesity phenotypes were defined as followed: 1) normal weight and normal metabolic status, 2) overweight and normal metabolic status, 3) obese and normal metabolic status, 4) normal weight and dysmetabolic status, 5) overweight and dysmetabolic status, and 6) obese and dysmetabolic status. The association between maternal obesity phenotypes and incidence of obesity in children was studied using Cox proportional regression hazard model.

**Results:**

In male offspring, the risk of incidence of obesity significantly increased in those with maternal obesity phenotypes including overweight/normal metabolic: 1.75(95% CI: 1.10–2.79), obese/normal metabolic: 2.60(95%CI: 1.51–4.48), overweight/dysmetabolic: 2.34(95%CI: 1.35–4.03) and obese/dysmetabolic: 3.21(95%CI: 1.94–5.03) compared to the normal weight/normal metabolic phenotype. Similarly, in girls, the risk of incidence of obesity significantly increased in offspring with maternal obesity phenotypes including overweight/normal metabolic: 2.39(95%CI: 1.46–3.90), obese/normal metabolic: 3.55(95%CI: 1.94–6.46), overweight/dysmetabolic: 1.92(95%CI: 1.04–3.52) and obese/dysmetabolic: 3.89(95%CI: 2.28–6.64) compared to normal weight/normal metabolic phenotype. However, maternal normal weight/dysmetabolic phenotype did not significantly change the risk of obesity in both male and female offspring.

**Conclusion:**

Except for normal weight/dysmetabolic phenotype, all maternal obesity phenotypes had significant prognostic values for incidence of offspring obesity with the highest risk for obese/dysmetabolic phenotype. This study provides valuable findings for identifying the first line target groups for planning interventions to prevent childhood obesity.

**Supplementary Information:**

The online version contains supplementary material available at 10.1186/s12889-021-10932-4.

## Introduction

The global prevalence of childhood obesity shows a rising trend over recent decades [1], with a relatively high prevalence of overweight and obesity reported in Iranian children. Based on data available, in a nation-wide study conducted in 2015 among 7–18 years Iranian students, approximately one-fifth of participants (20.8%) were found to be overweight and obese [2]. In Tehran, the prevalence rates of overweight and obesity ranged from more than 14% in preschool children (2011) to about 38% in adolescents (2014) [3, 4]. Overweight and obesity in children is not only associated with cardio-metabolic disorders such as type II diabetes, cardiovascular diseases (CVDs), hyperlipidemia, hypertension, and early atherosclerosis; it is also related to the development of other health complications such as respiratory diseases, musculoskeletal complaints and psychosocial problems [5–8]. Considering the remarkable proportion of children affected by obesity and its related health consequences of excessive weight [1–8], it is important to identify factors associated with the development of obesity in the early years of life. Different variables, including lifestyle factors such as levels of physical activity and sedentary behaviors, dietary intakes and eating habits, certain socio-environmental factors, including socio-economic status and parental factors, play a major role in weight gain during childhood and adolescence [9, 10].

The relationship between parental obesity and offspring weight status has been previously documented in different populations [11–13]. Similarly, studies conducted in Iran show that both paternal and maternal overweight/obesity are important determinants of overweight/obesity in children [14–16]. These studies were mainly focused on overweight/obesity, which is commonly defined using body mass index (BMI), but it should be kept in mind that BMI has some limitations which confine its ability to be a good measure of health risk. For instance, it does not necessarily reflect body fatness, nor does it indicate the distribution of body fat. The aforementioned issues limit its potential to be a good measure for obesity-related cardiometabolic risk factors. In this regard, existing evidence reports a higher risk for incidence of CVD outcomes among normal-weight individuals with the dysmetabolic condition compared to their obese counterparts without dysmetabolic condition [17]. In another study, the risk of CVD did not significantly differ between non-obese metabolically healthy and obese metabolically healthy participants; however, participants with two or more metabolic abnormalities were significantly at higher risk of CVD compared to non-obese metabolically healthy participants [18]. Moreover, findings of a meta-analysis, in obese individuals with unhealthy metabolic status, vascular function had more impaired than obese individuals with healthy metabolic status [19]. These findings highlight the decisive role of a metabolic condition in determining the cardiovascular health status of individuals. Therefore, researchers have begun using a new classification, viz. obesity phenotype, which categorizes individuals considering both their body weight and metabolic status [20]. Hence, to determine factors influencing health risk, it would be better and more informative to use obesity phenotypes rather than obesity per se.

Parental weight status is widely studied as an important determinant of childhood obesity [11–16]. In this regard, findings of a study conducted on participants of the Tehran Lipid and Glucose Study (TLGS) emphasized the importance of parental obesity in the incidence of obesity in 7–11 years children during 10 years follow up [21]. Although the role of parental obesity in childhood obesity has been well studied, obesity-related metabolic abnormalities have been less considered as determinants of childhood obesity. In another report, the synergic effect of several parental factors which could predispose offspring to be overweight/obese was investigated. Obtained results indicated among studied factors, maternal metabolic syndrome as well as age, education and body weight status were the most important factors in differentiating parental risk clusters which could predict incidence of overweight and obesity in offspring [15]. Although the mentioned study revealed both maternal metabolic and weight status as important modifiable factors which could predispose children to be overweight/obese; prognostic values of different maternal obesity phenotypes as a combination of maternal weight and metabolic status on childhood obesity is still unclear and needs more longitudinal investigations. A study measuring prognostic values of different maternal obesity phenotypes can provide more informative evidence to identify vulnerable groups of mothers which need to be targeted in future childhood obesity related interventions. Hence, this study for the first time aimed to investigate the prognostic value of maternal obesity phenotypes in development offspring obesity during more than a decade follow-up in a large cohort of the Tehranian population.

## Methods

### Participants and design

The current longitudinal study was conducted within the framework of the TLGS. The TLGS is a prospective study that has two major parts: 1) a cross-sectional prevalence study of non-communicable diseases (NCDs) and their associated risk factors; 2) an ongoing prospective follow-up study in which NCD risk factors are measured every 3 years. In brief, using the multistage cluster random sampling method, a total of 15,005 individuals aged ≥3 years were selected from three medical health centers under the coverage of Shahid Beheshti University of Medical Sciences in district 13 of Tehran (located in the east of Tehran). Details of the rationale and design of the TLGS have been published elsewhere [22, 23].

For the current longitudinal study, TLGS participants aged 3–19 years at baseline (1999 to 2001) and their mothers were chosen. Inclusion criteria for children were those who were not obese at baseline and had complete parental information. Since the main objective of the current study was to investigate incidence of obesity in offspring in the cohort, all obese children at baseline were excluded. Hence, of a total of 4351 participants aged 3–19 years at baseline, 558 children were excluded because of being obese or having missing data on BMI status at baseline. A further 1642 children were excluded because they lacked complete data on parental characteristics (*n* = 1122) or were lost to follow up (*n* = 520). Finally, 2151 children were remained and included in the analysis who were followed until the incidence of obesity. The ethics committee of the Research Institute for Endocrine Sciences approved this study (IR.SBMU.ENDOCRINE.REC.1395.256), and informed written consents were obtained from all participants. For participants less than 18 years of age/illiterates, informed consent was obtained from their parents /legally authorized representative.

### Measurements

Baseline assessments have been conducted during 1999–2001, and follow-up assessments have been repeated every 3 years.

#### Baseline measurements in parents

To obtain parental information, both fathers and mothers were interviewed by trained interviewers, and information on their own age, gender, education status, employment, physical activity, and smoking habit were gathered (Additional file 1). Physical activity level was assessed using a Lipid Research Clinic questionnaire [24] and categorized into three groups: high, moderate, and low physical activity, which was defined as exercising or having physical activity at least three times a week, exercising or having physical activity less than three times a week, and having no exercise or physical activity in the past week, respectively. Smoking status was categorized into two groups: 1) non-smokers and 2) current smokers (regular or irregular smokers).

In addition, anthropometric indices included weight, height, and waist circumference, were measured. Weight was measured, while participants were minimally clothed, without shoes, using digital scales and recorded to the nearest 100 g. Height was measured in a standing position without shoes, using a stadiometer, with shoulders in normal alignment. BMI was calculated as weight (kg) divided by the square of height (m^2^). Waist circumference was measured at the umbilical level, over light clothing, using a tape meter, without any pressure to the body surface, and measurements were recorded to the nearest 0.1 cm. Blood pressure was measured twice, after participants were seated for 15 min, using a standard mercury sphygmomanometer; there was at least 30s interval between these two separate evaluation, and the mean of two measurements was considered as the blood pressure [23]. A blood sample was taken after a 12 to 14-h overnight fasting. All blood analyses were conducted at the TLGS research laboratory on the day of blood collection. Further details on the assessment of fasting blood sugar (FBS) and serum lipids (high-density lipoprotein cholesterol (HDL-C) and triglycerides (TG)) have been provided previously [22]. Standard oral glucose tolerance tests were done for participants [22].

Maternal obesity phenotypes were defined using mothers’ BMI categories and metabolic status. Mothers’ BMIs were classified into three categories: BMI < 25 kg/m^2^ as normal weight, BMI ≥25 to 29.9 kg/m^2^ as overweight, and BMI ≥30 kg/m^2^ as obese [25]. Mothers’ metabolic status has been categorized into two groups: normal metabolic and dysmetabolic status. Dysmetabolic status was considered as having either metabolic syndrome or diabetes. Metabolic syndrome was defined according to the joint interim statement criteria, which determined as having at least three out of the following five criteria: 1) elevated waist circumference ≥ 90 cm in both gender [26], 2) reduced HDL-C (< 50 mg/dl in women, < 40 in men) or on drug treatment for reduced HDL-C, 3) elevated TG levels ≥150 mg/dl or on drug treatment for elevated TG, 4) elevated blood pressure (≥130 mm/hg systolic blood pressure or ≥ 85 mm/hg diastolic blood pressure) or on antihypertensive drug treatment in a patient with a history of hypertension and 5) elevated FBS ≥100 mg/dl or on drug treatment for elevated glucose [27]. Diabetes was defined according to the criteria of the American diabetes association as fasting plasma glucose ≥126 mg/dl or 2-h post 75-g glucose load ≥200 mg/dl or current therapy for a definite diagnosis of diabetes [28]. Considering three BMI categories (normal weight, overweight and obese) and two metabolic status (normal metabolic and dysmetabolic), six obesity phenotypes were defined. 1) normal weight and normal metabolic status, 2) overweight and normal metabolic status, 3) obese and normal metabolic status, 4) normal weight and dysmetabolic status, 5) overweight and dysmetabolic status, and 6) obese and dysmetabolic status [17].

#### Baseline measurements in children

Age, weight, and height were variables that have been collected for children. Children’s age was determined using the date of birth of children, which was reported by their parents. Children’s weight and height were measured as explained earlier, and their BMI was calculated as weight (kg) divided by the square of height (m^2^).

#### Outcome measurements

In the current study, offspring’s obesity has been considered as the outcome. In participants aged < 19 years, obesity has been defined using sex-specific BMI-for-age percentile curves developed by the World Health Organization [29]. Children and adolescents with a BMI > the 95th percentile were considered obese [29]. In participants ≥19 years, obesity has been defined as having BMI ≥30 kg/m^2^ [25]. The event date for incident cases of obesity was defined as the midpoint between the date of follow-up at which obesity was diagnosed for the first time and the most recent follow-up visit prior to diagnosis.

### Statistical analysis

All continuous data are expressed as mean ± stanadrad deviation, and categorical variables are expressed as frequency (percentages). Independent samples t-test, one-way ANOVA test, Fisher exact test, and Chi-square test were used to compare continuous and categorical variables between maternal phenotype groups. Children 3–19 years of age, who were not obese at baseline (1999 to 2001) were followed until the occurrence of obesity. Endpoints were considered as the date of incident obesity or censoring. Censoring was defined as leaving the residence area, death, lost to follow-up or end of follow-up. The event date for the incident cases of obesity was defined as the mid time between the date of follow-up visit at which obesity was diagnosed for the first time, and the most recent follow-up visit prior to the diagnosis and for those with negative event (censored subjects), the time was the interval between the first and the last observation dates. Duration between the end points and baseline assessment was considered as survival time for current study. Kaplan Meier survival curves were used to demonstrate occurrence of children’s obesity during the period of follow-up assessment by maternal obesity phenotypes. The association between maternal obesity phenotypes and incidence of obesity in children was assessed using Cox proportional-hazards models. In these models, offspring were stratified into six groups based on maternal obesity phenotypes and the group with maternal normal weight and normal metabolic status, was considered as the reference group. Unadjusted and adjusted hazard ratios of obesity event in children were calculated for maternal obesity phenotypes. The first model is an unadjusted model and model 2 is adjusted for children’s age at baseline. In model 3, except for children’s age, all parental variables that were significantly different among maternal obesity phenotype groups at baseline, were also adjusted. The proportionality assumption of Cox models was assessed using Schoenfeld residuals test and it was appropriate. SPSS version 15 was used for data analysis, and *p*-values < 0.05 were considered statistically significant.

## Results

At baseline, the mean age of children and mothers were 11.9 ± 4.4 and 38.4 ± 7.5 years, respectively. Descriptive statistics of mothers at baseline are presented in Table 1. The distribution of maternal obesity phenotypes was as follows: Normal weight/normal metabolic: 23.5%; overweight/normal metabolic: 29.7%; obese/normal metabolic: 9.2%; normal weight/dysmetabolic: 3.1%; overweight/dysmetabolic: 15.7% and obese/dysmetabolic: 18.8%. Descriptive statistics for age and BMI of offspring at baseline and follow-ups are presented as a supplementary table (Additional file 2). The incidence of obesity in male and female offspring was 16.73 (95%CI: 14.48–19.26) and 13.76 (95%CI: 11.84–15.99) per 1000 person-years, respectively.

**Table 1:** Descriptive statistics of mothers at baseline

Maternal factors^**a**^	Total
**Age (year)**	38.4 ± 7.5
**Education n(%)**	
-Illiterate & primary	701 (32.6)
-Secondary& higher	1450 (67.4)
**Employment status n(%)**	
-Employed	196 (9.1)
-Un-employed	1955 (90.9)
**Physical activity n(%)**	
-Low	1377 (64)
-Moderate or High	774 (36)
**BMI (kg/m2)**	27.9 ± 4.5
**Waist circumference (cm)**	87.5 ± 11.1
**FBS (mg/dl)**	95.4 ± 29.4
**2 h blood sugar (mg/dl)**	118.9 ± 52.5
**TG (mg/dl)**	132 (90–197)
**HDL-C (mg/dl)**	44.2 ± 10.8
**SBP (mm Hg)**	115.2 ± 15.3
**DBP (mm Hg)**	77.5 ± 10.0
**Diabetes (Yes) n(%)**	184 (8.6)
**Metabolic syndrome (Yes) n(%)**	791 (36.8)

*BMI* Body mass index, *FBS* Fasting blood sugar, *TG* Triglycerides, *HDL-C* High-density lipoprotein cholesterol, *SBP* Systolic blood pressure, *DBP* Diastolic blood pressure

^a^Data are presented as mean ± SD, except for education, employment status, physical activity, diabetes, and metabolic syndrome

Descriptive statistics of parents stratified by maternal obesity phenotypes are illustrated in Table 2; as indicated, maternal age (*p* < 0.001) and level of education (*p* < 0.001) were significantly different among maternal obesity phenotypes. In terms of father’s characteristics, paternal level of education (*p* < 0.001), employment status (*p* < 0.001), and body weight status (*p* < 0.05) were significantly different among maternal obesity phenotypes.

**Table 2:** Baseline characteristics of study participants by maternal obesity phenotypes

	Boys (*n* = 1037)							Girls (*n* = 1114)						
Maternal weight status^a^	N.W	OW.	Ob.	N.W	OW.	Ob.	*p*-value	N.W	OW.	Ob.	N.W	OW.	Ob.	*p*-value
Maternal metabolic status	Normal metabolic status			Dysmetabolic status				Normal metabolic status			Dysmetabolic status			
**Mothers’ characteristics**														
**Age**	35.1 ± 6.9	36.6 ± 6.6	37.2 ± 7.0	43.5 ± 7.1	42.0 ± 7.0	41.7 ± 7.0	< 0.001	35.4 ± 6.5	37.4 ± 6.9	37.8 ± 7.3	39.9 ± 8.2	41.8 ± 8.3	41.2 ± 7.0	< 0.001
**Physical activity**														
Low	143 (61.1)	187 (62.1)	62 (60.8)	16 (48.5)	110 (67.1)	134 (66.0)	0.348	179 (65.8)	215 (63.6)	60 (61.9)	26 (78.8)	116 (67.1)	129 (64.2)	0.560
Moderate or High	91 (38.9)	114 (37.9)	40 (39.2)	17 (51.5)	54 (32.9)	69 (34.0)		93 (34.2)	123 (36.4)	37 (38.1)	7 (21.2)	57 (32.9)	72 (35.8)	
**Employment status**														
Employed	24 (10.3)	34 (11.3)	7 (6.9)	2 (6.1)	17 (10.4)	8 (3.9)	0.072	33 (12.1)	38 (11.2)	4 (4.1)	3 (9.1)	14 (8.1)	12 (6.0)	0.073
Un-employed	210 (89.7)	267 (88.7)	95 (93.1)	31 (93.9)	147 (89.6)	195 (96.1)		239 (87.9)	300 (88.8)	93 (95.9)	30 (90.9)	159 (91.9)	189 (94.0)	
**Education**														
Illiterate & primary	62 (26.5)	65 (21.6)	36 (35.3)	14 (42.4)	71 (43.3)	93 (45.8)	< 0.001	54 (19.9)	81 (24.0)	32 (33.0)	16 (48.5)	82 (47.4)	95 (47.3)	< 0.001
Secondary& higher	172 (73.5)	236 (78.4)	66 (64.7)	19 (57.6)	93 (56.7)	110 (54.2)		218 (80.1)	257 (76.0)	65 (67.0)	17 (51.5)	91 (52.6)	106 (52.7)	
**Fathers’ characteristics**														
**Physical activity**														
Low	160 (68.4)	192 (63.8)	73 (71.6)	19 (57.6)	108 (65.9)	133 (65.5)	0.590	173 (63.6)	218 (64.5)	62 (63.9)	25 (75.8)	99 (57.2)	115 (57.2)	0.178
Moderate or High	74 (31.6)	109 (36.2)	29 (28.4)	14 (42.4)	56 (34.1)	70 (34.5)		99 (36.4)	120 (35.5)	35 (36.1)	8 (24.2)	74 (42.8)	86 (42.8)	
**Employment status**														
Employed	218 (93.2)	279 (92.7)	92 (90.2)	22 (66.7)	140 (85.4)	163 (80.3)	< 0.001	257 (94.5)	312 (92.3)	86 (88.7)	29 (87.9)	142 (82.1)	170 (84.6)	< 0.001
Un-employed	16 (6.8)	22 (7.3)	10 (9.8)	11 (33.3)	24 (14.6)	40 (19.7)		15 (5.5)	26 (7.7)	11 (11.3)	4 (12.1)	31 (17.9)	31 (15.4)	
**Education**														
Illiterate & primary	51 (21.8)	57 (18.9)	35 (34.3)	16 (48.5)	51 (31.1)	77 (37.9)	< 0.001	53 (19.5)	80 (23.7)	31 (32.0)	19 (57.6)	64 (37.0)	83 (41.3)	< 0.001
Secondary& higher	183 (78.2)	244 (81.1)	67 (65.7)	17 (51.5)	113 (68.9)	126 (62.1)		219 (80.5)	258 (76.3)	66 (68.0)	14 (42.4)	109 (63.0)	118 (58.7)	
**Smoking**														
Smoker	75 (32.1)	89 (29.6)	28 (27.5)	13 (39.4)	53 (32.3)	46 (22.7)	0.174	92 (33.8)	124 (36.7)	29 (29.9)	13 (39.4)	45 (26.0)	55 (27.4)	0.085
Non smoker	159 (67.9)	212 (70.4)	74 (72.5)	20 (60.6)	111 (67.7)	157 (77.3)		180 (66.2)	214 (63.3)	68 (70.1)	20 (60.6)	128 (74.0)	146 (72.6)	
**BMI status**														
Normal weight	114 (48.7)	116 (38.5)	29 (28.4)	10 (30.3)	55 (33.5)	71 (35.0)	0.013	135 (49.6)	131 (38.8)	31 (32.0)	14 (42.4)	60 (34.7)	88 (43.8)	0.029
Overweight	99 (42.3)	145 (48.2)	61 (59.8)	17 (51.5)	85 (51.8)	98 (48.3)		106 (39.0)	141 (41.7)	49 (50.5)	15 (45.5)	82 (47.4)	79 (39.3)	
Obese	21 (9.0)	40 (13.3)	12 (11.8)	6 (18.2)	24 (14.6)	34 (16.7)		31 (11.4)	66 (19.5)	17 (17.5)	4 (12.1)	31 (17.9)	34 (16.9)	

Data are presented as number (%) except for maternal age, which is reported as mean ± SD

^a^ *N.W.* Normal weight (BMI < 25 kg/m^2^), *OW.* Overweight (25 ≤ BMI < 30 kg/m^2^), *Ob.* Obese (≥30 kg/m^2^)

**Fig. 1:**
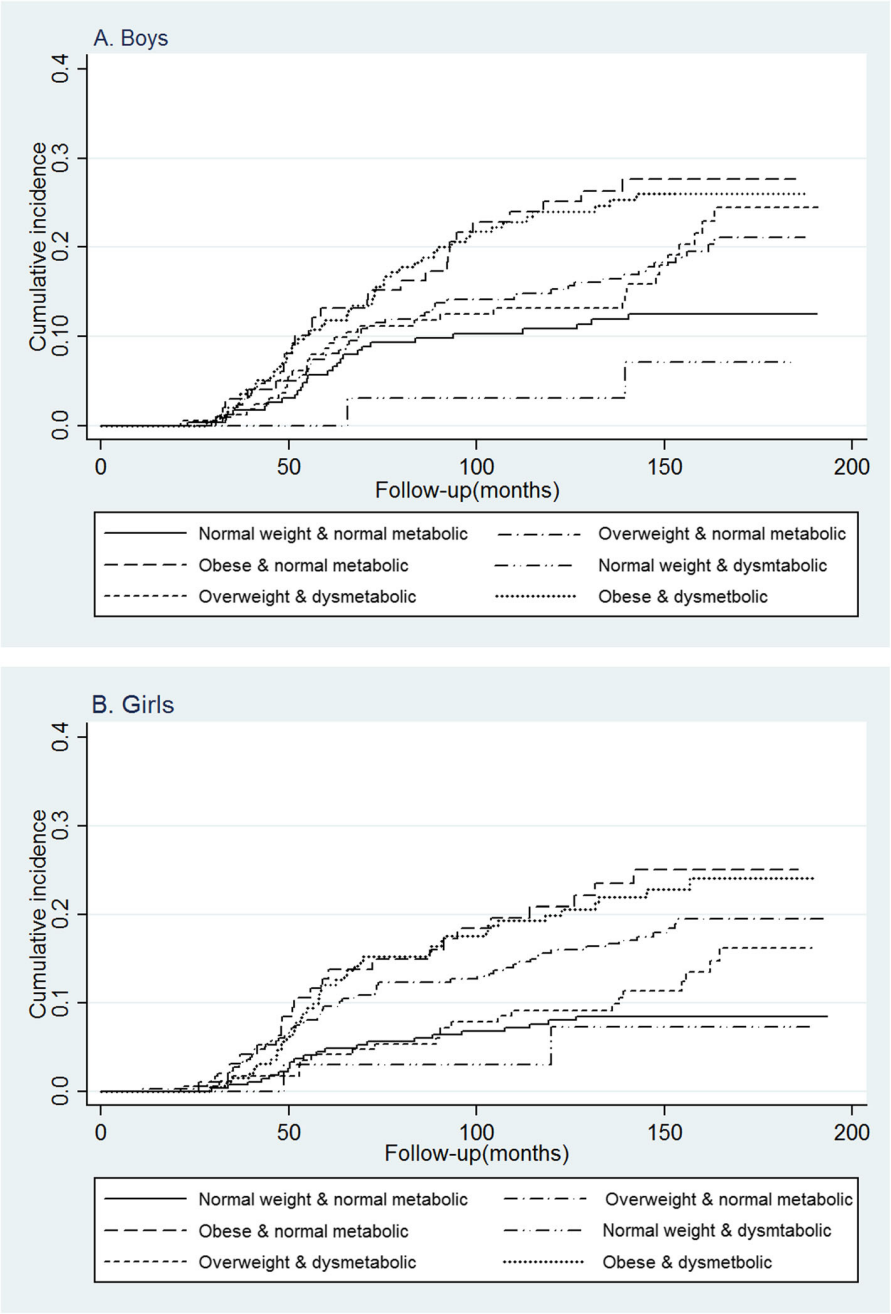
Kaplan-Meier curve of cumulative incidence for obesity in boys (**a**) and girls (**b**) according to maternal obesity phenotypes

Of a total of 2151 non-obese children at baseline, 359 (17.7%) new obesity event occurred after 148.7 ± 34.7 months of follow up. Kaplan Meier curves (Fig. 1) demonstrate that the incidence of obesity in both male and female offspring of mothers with various obesity phenotypes differed significantly (log-rank *p*-values were 0.003 and < 0.001, respectively, for male and female). In both male and female offspring, the highest incidence of obesity was observed in those whose mothers had obese-(dysmetabolic or normal metabolic) phenotypes, and the lowest incidence of obesity was observed in those whose mothers had normal weight/dysmetabolic phenotype.

Table 3 shows the estimated results of Cox proportional hazard models. For both boys and girls, the hazard ratios and 95% confidence intervals (CIs) of obesity incidence were calculated for different maternal obesity phenotypes. In all three models, compared to normal weight/normal metabolic phenotype, the risk of obesity in offspring was higher among all maternal obesity phenotypes, except for the normal weight/dysmetabolic phenotype. In the final adjusted model, compared to mothers with normal weight/normal metabolic phenotype, the risks of obesity were significantly higher in male offspring of mothers with overweight/normal metabolic phenotype, obese/normal metabolic phenotype, overweight/dysmetabolic phenotype, and obese/dysmetabolic phenotype. Similarly, compared to mothers with normal weight/normal metabolic phenotype, the risks of obesity were significantly higher in female offspring of mothers with the overweight/normal metabolic phenotype, obese/normal metabolic phenotype, overweight/dysmetabolic phenotype, and obese/dysmetabolic phenotype (Table 3).

**Table 3:** Hazard ratios of obesity incidence for maternal obesity phenotypes in boys and girls

	Obesity phenotype	Boys (***n*** = 1037)		Girls (***n*** = 1114)	
		HR(95%CI)	*p*-value	HR(95%CI)	*p*-value
**Model 1**	Normal weight/normal metabolic	1		1	
	Overweight/normal metabolic	1.59 (1.00–2.52)	0.048	2.32 (1.42–3.77)	0.001
	Obese/normal metabolic	2.36 (1.38–4.05)	0.002	3.19 (1.77–5.77)	< 0.001
	Normal weight/Dysmetabolic	0.48 (0.12–2.03)	0.321	0.78 (0.18–3.31)	0.735
	Overweight/Dysmetabolic	1.66 (0.99–2.78)	0.054	1.58 (0.88–2.86)	0.128
	Obese/Dysmetabolic	2.22 (1.39–3.56)	0.001	2.96 (1.77–4.96)	< 0.001
**Model 2**	Normal weight/normal metabolic	1		1	
	Overweight/normal metabolic	1.86 (1.17–2.96)	0.009	2.62 (1.60–4.28)	< 0.001
	Obese/normal metabolic	2.80 (1.63–4.81)	< 0.001	3.93 (2.16–7.13)	< 0.001
	Normal weight/Dysmetabolic	0.68 (0.16–2.85)	0.593	0.97 (0.23–4.15)	0.972
	Overweight/Dysmetabolic	2.25 (1.33–3.81)	0.003	2.08 (1.14–3.79)	0.016
	Obese/Dysmetabolic	2.98 (1.84–4.84)	< 0.001	3.83 (2.26–6.46)	< 0.001
**Model 3**	Normal weight/normal metabolic	1		1	
	Overweight/normal metabolic	1.75 (1.10–2.79)	0.018	2.39 (1.46–3.90)	0.001
	Obese/normal metabolic	2.60 (1.51–4.48)	0.001	3.55 (1.94–6.46)	< 0.001
	Normal weight/Dysmetabolic	0.78 (0.18–3.33)	0.738	0.94 (0.22–4.03)	0.932
	Overweight/Dysmetabolic	2.34 (1.35–4.03)	0.002	1.92 (1.04–3.52)	0.036
	Obese/Dysmetabolic	3.21 (1.94–5.30)	< 0.001	3.89 (2.28–6.64)	< 0.001

Model 1: Unadjusted, Model 2: Adjusted for child’s age, Model 3: Adjusted for child’s age, mother’s (age & education), and father’s (education, job status & BMI status). BMI classification values: Normal weight (BMI < 25 kg/m^2^), Overweight (25 ≤ BMI < 30 kg/m^2^), Obese (≥30 kg/m^2^)

## Discussion

Findings of this prospective study conducted on an urban Iranian population indicate that after adjusting potential confounders, except for normal weight/dysmetabolic phenotype, all maternal obesity phenotypes significantly increased the risk of incidence of obesity in offspring compared to the normal weight/normal metabolic phenotype, findings implying the prognostic value of maternal body weight status rather than of their metabolic status in the development of overweight and obesity in children.

Current findings indicate the important role of maternal overweight and obesity in the development of overweight/obesity in their offspring, a finding in line with those of previous studies highlighting the important role of both maternal and paternal overweight/obesity in childhood obesity in Iran [15, 30, 31] as well as in other countries [12, 13, 32]. Previous studies highlight the role of both parents’ weight status in the development of childhood obesity; however, they could not come to an agreement regarding the importance of parent’s weight status in the development of obesity in children. Some studies indicate a stronger association between maternal overweight/obesity and childhood obesity, especially at birth and infancy compared to paternal overweight/obesity [33–35]. Nevertheless, there are studies indicating paternal overweight/obesity tends to show their effects later [36], and hence, its effect should not be neglected. In our previous analysis, we considered multiple parental risk factors to identify groups at higher risk for childhood obesity, and findings revealed that maternal characteristics played a more important role compared to paternal characteristics [15]; therefore, in the current study, we mainly focused on maternal characteristics. However, as the role of paternal characteristics in childhood obesity should not be ignored, we adjusted the father’s characteristics in the final model.

In the current study, the coincidence of maternal overweight/obesity and dysmetabolic status did not increase the risk of incidence of childhood obesity compared to maternal overweight/obesity per se. Furthermore, dysmetabolic status in normal-weight mothers did not significantly increase the rate of obesity in their offspring. Previous findings of the TLGS population indicates that among various obesity phenotypes, only obese women with dysmetabolic status perceived poor physical health status compared to women with normal weight-normal metabolic phenotypes [37]. Therefore, it was reasonable to assume that obesity risk perception among mothers with cardio-metabolic risk factors (dysmetabolic status) could be higher than those without these risk factors; therefore, we expected lower rates of obesity in the offspring of these mothers. However, the current findings did not confirm our hypothesis, suggesting that perceived threat by overweight/obese mothers with dysmetabolic status is similar to those mothers who are only overweight/obese, i.e., mothers’ perceptions of their risk status do not necessarily raise concerns about their child’s weight status. Therefore, considering existing evidence regarding the association between risk perception and health-related behaviors [38, 39] and the prominent role of mothers in shaping their offspring lifestyle through creating a supportive home environment, role modeling, parenting style feeding, and activity practices [40–47]; targeting all overweight/obese mothers irrespective of their metabolic status to improve their risk perception regarding excessive weight gain in their children would be recommended. Since overweight and obese mothers who do not have metabolic disorders are less likely to go to health centers due to lack of metabolic complications; therefore, educational interventions aimed at preventing weight gain in offspring of this group of mothers is highlighted.

In the current study, there were no sex differences in the pattern of association between maternal obesity phenotypes and incidence of obesity in offspring. In terms of sex differences in the association between weight and metabolic status of parents and obesity in their offspring, existing evidence is mixed. Previous evidence revealed a sex-based difference in the prevalence of childhood obesity due to the existence of differences in the body composition, patterns of weight gain, hormone biology, bodyweight ideals, and susceptibility to socio-environmental factors of boys and girls [48]. In addition, in some previous studies which examined the association between parental factors and offspring weight status, a sex-specific pattern was evident [49, 50]. However, the findings of the current study did not reveal any sex-specific pattern in the findings. In agreement with the current findings, a cross-sectional study in Germany demonstrated a negligible difference in association between parental and offspring’s BMIs in boys and girls [51]. While findings of a study on Canadian children aged 6–10 years showed stronger association between parental and offspring BMIs in girls, compared to boys [49]; another study in the United States revealed a significant association between gestational diabetes mellitus as a maternal metabolic disorder and offspring obesity, only in boys [50]. However, mentioned studies did not focus on both parental weight and metabolic features simultaneously; this issue restricted comparison of the current findings with previous findings. Therefore, to replicate current findings, more longitudinal studies investigating the synergic effects of parental weight and metabolic status on incidence of obesity in offspring seems essential.

The current study has both strengths and limitations; its large sample size, the longitudinal nature of the study with long-term follow up, and objective measurements of weight and height of participants are among the main strengths. Another strength is considering several important confounding variables in the analysis, including age and sex of children, parental age, educational level, job status, and father’s BMI. Limitations of this study also need to be mentioned. First, considering childhood obesity as a multi-factorial disorder that is influenced by several factors, all potential associated factors, including genetic and lifestyle factors, were not considered in the current study, and it is recommended they be considered in future studies to provide more accurate findings. Second, all participants recruited for the TLGS were ≥ 3 years. Data of children less than 3 years was not available in the current analysis. It may influence the findings of the current study; therefore, in future studies, it is recommended to consider collecting participants’ data from birth. In addition, dysmetabolic women tend to have excessive weight. Therefore, the prevalence of normal weight/dysmetabolic phenotype in mothers was low in the current study. The small number of normal weight/dysmetabolic phenotype mothers, limited statistical power of the current analysis, which might be a reason of the non-significant association of normal weight/dysmetabolic phenotype with children’s obesity. Moreover, in the current study, if we stratified children’s age, we would not have a sufficient number of participants in some subgroups of maternal obesity phenotypes. Also, maternal obesity phenotypes may affect the obesity of their children differently in the course of children’s growth. Therefore, future studies are recommended to analyze the association between maternal obesity phenotypes and children’s obesity separately in the childhood and adolescent stages. Finally, the participants of the TLGS are confined to Tehranian population; hence, our results cannot be generalized to all Iranian populations, especially those residing in the sub-urban and rural areas.

## Conclusion

In conclusion, all maternal obesity phenotypes had significant prognostic values for incidence of offspring obesity, except for normal weight/dysmetabolic phenotype. Findings highlight the necessity of healthy lifestyle interventions to improve weight and metabolic status in mothers as an important target group to prevent childhood obesity.

## Supplementary Information

**Additional file 1.** Socio-demographic and behavioral questions.

**Additional file 2: Table S1.** Descriptive statistics of offspring at baseline and follow-ups.

## Data Availability

The datasets used and/or analyzed during the current study are available from the corresponding author on reasonable request.

## Abbreviations

BMI: Body mass index,
CVD: Cardiovascular disease
FBS: Fasting blood sugar
HDL-C: High-density lipoprotein cholesterol
NCDs: Non-communicable diseases
TG: Triglycerides
TLGS: Tehran Lipid and Glucose Study
