# Supplementary material for: Prognostic value of different maternal obesity phenotypes in predicting offspring obesity in a family-based cohort study
Source: BMC Public Health. 2021 May 8;21:885. doi: 10.1186/s12889-021-10932-4 (PMC8106828; doi:10.1186/s12889-021-10932-4)
Supplement: Supplementary file 1 — Additional file 1. Socio-demographic and behavioral questions. [file 12889_2021_10932_MOESM1_ESM.docx]

**Prognostic value of different maternal obesity phenotypes in predicting offspring obesity in a family-based cohort study**

Sara Jalali-Farahani^1, 2^, Parisa Amiri^1^, Bita Lashkari^1^, Leila Cheraghi^3^, Farhad Hosseinpanah^4^, Fereidoun Azizi^5^

**Affiliations:**

^1^Research Center for Social Determinants of Health, Research Institute for Endocrine Sciences, Shahid Beheshti University of Medical Sciences, Tehran, Iran.

^2^Students' Research Committee, Shahid Beheshti University of Medical Sciences, Tehran, Iran.

^3^Department of Epidemiology and Biostatistics, Research Institute for Endocrine Sciences, Shahid Beheshti University of Medical Sciences, Tehran, Iran.

^4^Obesity Research Center, Research Institute for Endocrine Sciences, Shahid Beheshti University of Medical Sciences, Tehran, Iran.

^5^Endocrine Research Center, Research Institute for Endocrine Sciences, Shahid Beheshti University of Medical Sciences, Tehran, Iran.

**Corresponding address:**

Parisa Amiri, PhD

Research Center for Social Determinants of Health,

Research Institute for Endocrine Sciences,

Shahid Beheshti University of Medical Sciences

P.O.Box: 19395-4763, Tehran, I. R. Iran,

Tel: +98 21 22432500, Fax: +98 21 22402463,

Email address: [amiri@endocrine.ac.ir](mailto:amiri@endocrine.ac.ir)

Questions used for definition of variables for the current study:

**Socio-demographic characteristics**

**Age:**

**Sex:** 1- Male □ 2- Female □

**Marital status:** 1- Single □ 2- Married □ 3- Divorced □ 4- Widowed □

**Education:**

**Literacy:** 1- Illiterate □ 2- Literate □

**What is your highest educational certificate?**

1- Primary School □ 2- 6-8th grade □ 3- 9-11th grade □ 4- High school □

5- Technician □ 6- Bachelor □ 7- Master □ 8- General practitioner □

9- Specialist □ 10- Nehzat □ 11- Religious Sciences □

**What has your occupational status been during the past month (>10 years old)?**

1- Employed □ 2- Unemployed □ 3- Student □ 4- Housewife □

5- Unemployed, having income □ 6- Others □

**Smoking**

**Do you smoke cigarettes?**

1- Yes, regularly □ 2- No □ 3- Yes, irregularly □

**Physical activity**

**Do you regularly engage in strenuous activities or hard physical labour?**

1- Yes □ 2- No □

**Do you exercise or labour at least three times a week?**

1- Yes □ 2- No □
