# Supplementary material for: Prognostic value of different maternal obesity phenotypes in predicting offspring obesity in a family-based cohort study
Source: BMC Public Health. 2021 May 8;21:885. doi: 10.1186/s12889-021-10932-4 (PMC8106828; doi:10.1186/s12889-021-10932-4)
Supplement: Supplementary file 2 — Additional file 2: Table S1. Descriptive statistics of offspring at baseline and follow-ups. [file 12889_2021_10932_MOESM2_ESM.docx]

**Prognostic value of different maternal obesity phenotypes in predicting offspring obesity in a family-based cohort study**

Sara Jalali-Farahani^1, 2^, Parisa Amiri^1^, Bita Lashkari^1^, Leila Cheraghi^3^, Farhad Hosseinpanah^4^, Fereidoun Azizi^5^

**Affiliations:**

^1^Research Center for Social Determinants of Health, Research Institute for Endocrine Sciences, Shahid Beheshti University of Medical Sciences, Tehran, Iran.

^2^Students' Research Committee, Shahid Beheshti University of Medical Sciences, Tehran, Iran.

^3^Department of Epidemiology and Biostatistics, Research Institute for Endocrine Sciences, Shahid Beheshti University of Medical Sciences, Tehran, Iran.

^4^Obesity Research Center, Research Institute for Endocrine Sciences, Shahid Beheshti University of Medical Sciences, Tehran, Iran.

^5^Endocrine Research Center, Research Institute for Endocrine Sciences, Shahid Beheshti University of Medical Sciences, Tehran, Iran.

**Corresponding address:**

Parisa Amiri, PhD

Research Center for Social Determinants of Health,

Research Institute for Endocrine Sciences,

Shahid Beheshti University of Medical Sciences

P.O.Box: 19395-4763, Tehran, I. R. Iran,

Tel: +98 21 22432500, Fax: +98 21 22402463,

Email address: [amiri@endocrine.ac.ir](mailto:amiri@endocrine.ac.ir)

**Table S1. Descriptive statistics of offspring at baseline and follow-ups**

|  | **Baseline** | **Follow up 1** | **Follow up 2** | **Follow up 3** | **Follow up 4** |
| --- | --- | --- | --- | --- | --- |
|  | **Age (year)** | | | | |
| **Boys** | 11.8±4.4 | 15.4±4.5 | 18.4±4.7 | 22.1±4.7 | 25.5±4.7 |
| **Girls** | 11.9±4.4 | 15.7±4.4 | 18.5±4.5 | 22.1±4.7 | 25.5±4.6 |
|  |  | | | | |
|  | **BMI (kg/m^2^)** | | | | |
| **Boys** | 17.5±3.0 | 20.2±3.8 | 22.2±4.0 | 23.8±4.2 | 25.0±4.0 |
| **Girls** | 17.9±3.5 | 20.4±3.8 | 21.9±3.8 | 23.5±4.1 | 24.3±4.3 |
